# Role of High-Density Lipoprotein Cholesterol (HDL-C) as a Clinical Predictor of Decompensation in Patients with Chronic Liver Disease (CLD)

**DOI:** 10.1155/2021/1795851

**Published:** 2021-12-24

**Authors:** Harshavardhan Rao B, Priya Nair, Anoop K. Koshy, S. Krishnapriya, C. R. Greeshma, Rama P. Venu

**Affiliations:** ^1^Department of Gastroenterology, Amrita Institute of Medical Sciences, Kochi, Kerala 682041, India; ^2^Healthcare Research Analyst, Department of Gastroenterology, Amrita Institute of Medical Sciences, Kochi, Kerala 682041, India; ^3^Department of Biostatistics, Amrita Institute of Medical Sciences, Kochi, Kerala 682041, India

## Abstract

**Introduction:**

Systemic inflammation triggered by bacterial products like lipopolysaccharides (LPS) in the circulation is an important factor leading to decompensation in patients with chronic liver disease (CLD). High-density lipoprotein cholesterol (HDL-C) has a significant role in innate immune response to LPS in the circulation and could therefore increase the risk for decompensation in patients with CLD. In this study, we have explored the role of HDL-C as a prognostic marker for decompensation.

**Methods:**

This was a prospective, observational, cohort study where consecutive patients with CLD were included. Patients with cholestatic liver disease and hepatocellular carcinoma were excluded. Fasting lipids were measured in all patients at the time of recruitment. Each patient was carefully followed up for development of decompensation events such as new-onset/worsening ascites, hepatic encephalopathy, or variceal bleed during follow-up.

**Results:**

A total of 170 patients were included (mean age 60 ± 11.5 years, M : F = 6 : 1). At the end of follow-up, 97/170 patients (57%) had decompensation events. Mean HDL-C levels were significantly lower among patients with decompensation (27.5 ± 15 mg/dL vs. 43.5 ± 13.9 mg/dL; *p* value 0.004). Using ROC analysis, cut-off for HDL-C of 36.4 mg/dL was identified. On multivariate analysis, HDL-C (OR = 6.072; 95% CI 2.39-15.39) was found to have an independent association with risk of decompensation.

**Conclusions:**

HDL-C level (<36.4 mg/dL) is a reliable marker for risk of decompensation and can be a useful addition to existing prognostic scoring systems in CLD. It can be a valuable tool to streamline treatment protocols and prioritise liver transplantation.

## 1. Introduction

The clinical spectrum of chronic liver disease (CLD) encompasses a clinically distinct compensated state (cCLD) to a symptomatic decompensated state (dCLD) and finally to the clinical syndrome of acute-on-chronic liver failure (ACLF) which is associated with multiple organ failure and a higher risk of mortality [1–4]. Decompensated chronic liver disease (dCLD) is associated with significant morbidity and mortality irrespective of etiology [1, 5]. dCLD is characterized by one or more of the following: variceal bleed, ascites, hepatic encephalopathy, or severe jaundice [4]. Factors that drive the progression of CLD are currently an area of intense research which can pave the way to develop a scoring system that can streamline treatment protocols. Currently used tools for prognosis like model for end-stage liver disease (MELD) [6] and Child-Pugh scores [7] lack the ability to accurately identify the dynamic clinical state and progression of CLD [8]. Recent studies have focussed on complex extrahepatic mechanisms that play a major role in the pathogenesis of decompensation in CLD. Among these, a systemic inflammatory process has been described, which has been found to be a major factor that results in decompensation [3, 9–11]. Systemic inflammation (SI) is triggered primarily by increased circulating bacterial products like lipopolysaccharides (LPS) and various pathogen-associated molecular patterns (PAMP). This has been attributed to quantitative and qualitative changes in the intestinal microbiome in patients with dCLD [12–14]. Interestingly, elevated inflammatory mediators as a part of SI have been shown to correlate with the risk of decompensation, ACLF, and poor clinical outcomes in patients with CLD [10, 11, 15].

High-density lipoprotein cholesterol (HDL-C) is a protein-rich lipoprotein that has been shown to have a role in reverse cholesterol transport, which essentially transports excess cholesterol from peripheral vessels to the liver for disposal, thereby reducing the risk of severe atherosclerotic disease [16]. The biochemical properties and metabolic pathway of HDL-C have uncovered additional anti-inflammatory properties especially in the setting of chronic systemic inflammatory disorders [17]. The structure of HDL is largely made up of two subclasses of particles—HDL2 and HDL3. While the larger HDL2 is involved in cholesterol transport, HDL3 particles are small, dense particles that have shown anti-inflammatory properties and can potentially attenuate the inflammatory response [18]. In addition, HDL3 has been demonstrated to have the ability to bind to and aid in excretion of bacterial lipopolysaccharides in the circulation [19]. In addition, HDL-C has also been shown to have distinct functional roles not just in lipid homeostasis but also in innate immunity where it has been closely linked with components of complement pathway and other proteins involved in immune function and the acute phase response [20]. This has led to studies that have delineated the role of these pleiotropic lipoproteins in the setting of various inflammatory disease conditions like rheumatoid arthritis, diabetes mellitus, and chronic kidney disease [21–23]. Patients with diseases characterized by a chronic systemic inflammatory response (diabetes mellitus, metabolic syndrome, and nonalcoholic fatty liver disease) and morphological and functional alterations that reduce the anti-inflammatory properties of HDL-C have been demonstrated [24, 25].

In the setting of CLD, enzymes required for maturation and metabolism of HDL have been shown to be markedly reduced [17]. In addition, altered serum levels of lipoproteins in CLD can be attributed to complex abnormalities in lipoprotein secretion, synthesis, and metabolism [26, 27]. In fact, lipoprotein alterations have been shown to be proportional to severity of liver disease [28]. Native gel analysis in patients with CLD has shown a shift towards HDL2 subclass with a marked reduction in HDL3 particles [29]. These findings allude to a functional impairment of HDL in addition to a quantitative reduction which can have a significant impact on the inflammatory response in patients with CLD, thereby indicating the risk of decompensation and progression to ACLF. In the background of these elegant studies which have identified HDL-C as a potential serum prognostic marker, we have measured the serum levels of HDL-C in a prospective cohort of patients with CLD, in order to test its efficacy in predicting decompensation events and short-term outcomes.

## 2. Materials and Methods

### 2.1. Study Design and Patients

This was a prospective, observational, cohort study conducted at a high-volume hepatology centre between January 2019 and December 2020. All consecutive patients diagnosed with CLD during the study period were considered for the study. A total of 240 consecutive patients diagnosed with CLD (based on clinical, biochemical, radiological, and/or histologic criteria) irrespective of etiology were recruited for the study. Liver transplant recipients and patients with hepatocellular carcinoma were excluded from the study. Patients with cholestatic liver diseases were also excluded owing to the effect of cholestasis on the serum lipids [30]. In addition, patients with other malignancies like intrahepatic cholangiocarcinoma and metastatic liver disease were also excluded from the analysis. After exclusion, a total of 170 patients were included after providing a written informed consent for participation. The study was approved by the institutional ethics committee and was conducted in accordance with the principles of the Declaration of Helsinki.

All relevant clinical data include demographics (age at diagnosis, gender, and underlying comorbidities), relevant radiological/histological reports, and endoscopy reports. Relevant laboratory values at recruitment and each follow-up were meticulously recorded and included tests of liver dysfunction (total bilirubin, albumin, prothrombin time, and liver enzymes), renal functions (serum creatinine and electrolytes), and markers of inflammation (leucocyte counts, neutrophil-lymphocyte ratio, and C-reactive protein levels). Routinely used prognostic scores like Child–Pugh (CP) classification score and MELD score were calculated for each patient at entry into the study.

### 2.2. Lipid Profile

A fasting blood sample was obtained from all patients included in the study (*n* = 170) within 48 hours of presentation to the hospital. Total cholesterol (TC), low-density lipoprotein (LDL), HDL-C, triglycerides (TG), and very low-density lipoprotein (VLDL) levels were then estimated (Hitachi 704 Analyzer—serviced by Roche Diagnostics) and recorded separately.

### 2.3. Follow-Up and Study Outcomes

All patients were followed up for a minimum of 1 year. At each visit, evidence for decompensation, laboratory evaluation (liver function tests, complete blood counts, electrolytes, renal function tests, prothrombin time/INR, and alpha fetoprotein), and ultrasound imaging of the abdomen were done. Acute decompensation/ACLF was managed as per institution protocol after hospital admission. Primary outcome of the study during follow-up was decompensation events, which was defined by the presence of at least 1 of the following: new-onset ascites/recurrence of ascites despite treatment, development of new-onset overt hepatic encephalopathy, development of spontaneous bacterial peritonitis, hepatorenal syndrome, or variceal bleeding requiring endoscopic variceal ligation/sclerotherapy. ACLF was defined as any patient who presented with acute decompensation and organ/system failure during follow-up [31]. In addition, liver transplantation and mortality at one year were also assessed as secondary outcomes.

### 2.4. Statistical Analysis

All statistical data analysis was carried out using IBM SPSS v20.0. Descriptive analysis of patients with decompensation events and stable decompensated disease was expressed as frequency and percentage for categorical variables and mean ± SD and median (Q1-Q3) for continuous variables. Area under ROC curve analysis was used to obtain an optimal cut-off value of HDL-C, with respect to decompensation events on follow-up. To test the statistical significance of categorical variables between the two outcome groups, chi-square with Fisher's exact test was applied. Statistical significance of continuous variables was computed by comparing the mean or median difference between groups, using independent sample *t*-test for parametric data and Mann Whitney *U* test for nonparametric data expressed as median (max-min). Independent association of the variables with the primary outcome (decompensated events) was assessed using multivariate backward conditional logistic regression analysis. All statistical tests were two-sided and conducted in an explorative manner on a significance level of <0.05.

## 3. Results

### 3.1. Baseline Characteristics of the Study Population at the Time of Subject Recruitment

A total of 170 patients were included in the study with a mean age of 60 ± 11.5 years and a male to female ratio of 6 : 1. The most common etiology of CLD was cryptogenic (85/170 patients (50%)) followed by alcohol-related CLD (74/170 patients (43.5%)). Viral hepatitis accounted for only 11/170 patients (6.4%). Patients with diabetes mellitus accounted for 110/170 patients (64.7%), and chronic kidney disease was seen in 30/170 patients (17.6%). Coronary artery disease was noted in 19/170 patients (11.2%) (see Table 1).

Child A status of liver disease was noted in 37/170 (21.8%) patients, while Child B status was seen in 32/170 patients (18.8%). Majority of patients were diagnosed with Child C disease which was noted in 101/170 patients (59.4%) at the time of recruitment into the study. The mean MELD score of the study population was found to be 20 ± 7.04. An upper endoscopy was done in all patients of the study. A total of 126/170 patients (74.11%) had esophageal/gastric varices, with 41/170 patients (24.11%) having a history of variceal bleeding that required endoscopic band ligation/sclerotherapy. All the patients were asymptomatic with no acute decompensation features at the time of recruitment for the study. A total of 95/170 patients (55.88%) had past history of ascites which was managed with diuretics and salt restriction, while 44/170 patients (25.88%) had at least one episode of hepatic encephalopathy in the past. However, at the time of recruitment, none of the patients had hepatic encephalopathy. The mean neutrophil-lymphocyte ratio (NLR) for the population was found to be 3.63 ± 3.93. All baseline demographic data have been presented in Table 1.

### 3.2. Clinical Course on Follow-Up

All patients were followed up for a minimum period of one year. Decompensation events were noted in 97/170 patients (57.05%) during follow-up. The most common decompensation event was ascites in 67/97 patients (69.07%) followed by hepatorenal syndrome in 45 patients (46.39%). Spontaneous bacterial peritonitis was diagnosed in 6/97 patients (6.18%) while 20/97 patients (20.61%) presented with a variceal bleed. Hepatic encephalopathy was diagnosed in 41/97 patients (42.26%). Bacterial sepsis was seen in 20/97 patients (20.61%) during follow-up. ACLF was diagnosed in 19/97 patients at the end of follow-up, with a mean CLIF-ACLF score of 67.57 ± 6.43. Among the patients with ACLF, 7/19 patients (36.9%) had a liver transplantation. Overall mortality at one year was found to be 43/170 patients (25.3%).

### 3.3. HDL-C with Severity of Liver Disease

HDL-C levels were measured at the time of recruitment for the study. Mean HDL-C levels were found to be 34.44 ± 16.57 mg/dL (median 34.75 mg/dL, range 4.6-78.7 mg/dL). Mean HDL-C was significantly lower in patients with Child C disease (31.25 mg/dL) as compared to patients with Child B (34.79 mg/dL) and Child A status of liver disease (42.84 mg/dL) (*p* value < 0.001). In addition, among patients with MELD < 10, mean HDL–C was found to be 45.13 ± 14.41 mg/dL as compared to the mean HDL-C of 33.09 ± 16.38 mg/dL among patients with MELD score > 10 (*p* value 0.002).

### 3.4. HDL-C as a Predictor of Clinical Outcomes

**Figure 1:**
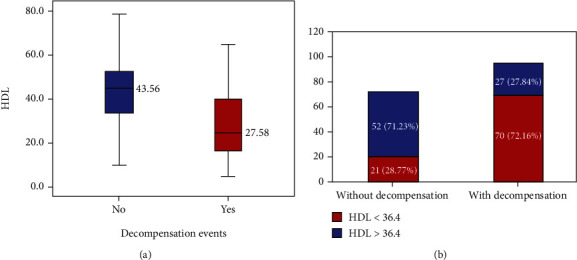
Association of HDL-C with decompensation events on follow-up in patients with CLD. (a) Comparison of mean HDL-C levels between patients with decompensation and those with stable decompensated disease. (b) Association of HDL − C < 36.4 mg/dL with risk of decompensation events on follow-up. ^∗^Abbreviations: CLD: chronic liver disease; HDL-C: high-density lipoprotein cholesterol.

Mean HDL-C was found to be significantly lower in patients with decompensation events (27.58 ± 15.07 mg/dL) as compared to those without decompensation during follow-up (43.56 ± 13.94 mg/dL) (*p* value 0.004) (see Figure 1). The area under ROC curve for HDL-C as a predictor for decompensation was found to be 0.782 (*p* value < 0.001) Using the coordinates on the curve, a cut-off of 36.4 mg/dL with a sensitivity of 72.16% and specificity of 71.23% was identified.

**Figure 2:**
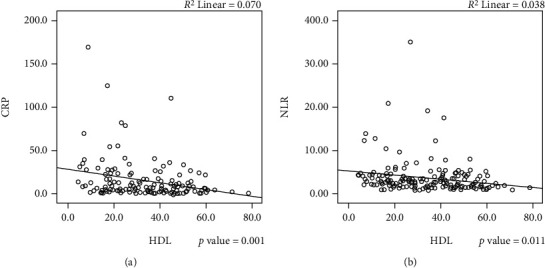
HDL-C levels showing a significant linear inverse correlation with markers of systemic inflammation: (a) correlation of HDL-C levels with CRP values (*p* value 0.001). (b) Correlation of HDL-C levels with NLR (*p* value 0.011). ^∗^Abbreviations: HDL-C: high-density lipoprotein cholesterol; CRP: C-reactive protein; NLR: neutrophil-lymphocyte ratio.

Among patients who had decompensation during follow-up, patients with HDL − C < 36.4 mg/dL accounted for 70/97 patients (72.2%) as compared to 21/73 patients (28.8%) among patients who have stable disease on follow-up (*p* value < 0.001) (see Figure 1). Apart from HDL-C, mean age (*p* value < 0.001), MELD score (*p* value < 0.001), Child status (*p* value < 0.001), diabetes mellitus (*p* value 0.004), serum albumin (*p* value < 0.001), INR (*p* value < 0.001), NLR (*p* value 0.003), sodium (*p* value 0.029), platelet count (*p* value 0.03), and total leucocyte counts (*p* value 0.048) were found to have a significant association with decompensation events during follow-up (see Table 2). However, multivariate analysis showed only HDL-C (OR = 6.072; 95% CI 2.39-15.39) and Child Status (OR = 4.541; 95% CI 1.17-17.57) were found to have an independent association with a risk of decompensation during follow-up. Mean HDL-C was found to be lower among patients who had died during follow-up (30.18 vs. 35.88, *p* value 0.05) with 26/43 patients (60.46%) who had HDL − C < 36.4. HDL-C was also found to be inversely related to inflammatory markers CRP (*p* value 0.001) and NLR in this study (*p* value 0.011) (see Figure 2).

## 4. Discussion

Decompensation in CLD can present with either ascites, variceal haemorrhage, hepatic encephalopathy, or jaundice [4]. Decompensation events, once developed, recur with increasing frequency, and most patients die within a median period of 2 years [1]. The course of dCLD is often complicated by the development of ACLF, a clinical syndrome characterized by acute deterioration with the development of extrahepatic organ failure. ACLF carries a high short-term mortality [31]. Several laboratory parameters and prognostic scoring systems have been studied to predict mortality in patients with dCLD. Among these, the Child-Pugh score has been extensively validated and comprises degree of ascites, prothrombin time, serum levels of bilirubin and albumin, and severity of hepatic encephalopathy. The MELD score is widely used in the transplant setting and comprises international normalized ratio in addition to serum levels of creatinine and bilirubin [32]. Although these scoring systems provide valuable information on the global liver dysfunction, they lack the ability to accurately identify the dynamic state of CLD ranging from compensated CLD, stable decompensated CLD to acute decompensation and pre-ACLF states [3, 4]. Early identification of these clinical states could potentially identify patients with poor outcomes in order to prioritise patients for LT.

One of the main drivers of decompensation has been found to be systemic inflammation (SI). Multiple studies have documented elevated levels of inflammatory molecules in patients with dCLD and correlated these to clinical outcomes [10, 11, 33]. Moreover, bacterial infections are twice as common in cirrhosis as compared to general population and are associated with significant mortality [34, 35]. SI in CLD can be attributed to increased bacterial translocation from the intestine secondary to a dysbiotic microbiome in conjunction with immune dysfunction that typically worsens with progression of liver disease and eventually leading to ACLF [36, 37]. HDL-C is a heterogenous particle of varying components that has been shown to have an immunomodulatory function. In fact, HDL-C has been shown to lose its anti-inflammatory properties in the setting of acute phase reactions [38]. However, elevated levels of HDL-C have not shown a protective role which indicates that associated proteins like apo-lipoproteins and functional alterations (like enzymatic activity) may play a significant role in its anti-inflammatory actions [17, 38]. Even in the setting of coronary artery disease, elevated HDL-C has not been shown to be protective and delineation of the exact role and mechanism of HDL-C in pathological processes needs further study [39]. In patients with CLD, the ability of HDL-C to suppress the levels of proinflammatory cytokines induced by bacterial LPS is impaired [17]. This indicates that low levels of HDL-C can play an important role in propagating SI in patients with dCLD. In a recent study, HDL-C and Apo-A1 were found to be MELD independent predictors of 90-day mortality [40]. In another retrospective study, HDL-C was found to be an independent predictor of short-term mortality in patients with CLD and variceal bleed [41].

In this exploratory prospective cohort study, HDL-C was found to have a significant correlation with known prognostic scoring systems like Child-Pugh and MELD scores. More importantly, serum HDL-C levels were found to be independently associated with the risk of decompensation in patients with CLD. The area under ROC for decompensation was found to be 0.782 (*p* value < 0.001). Patients with HDL − C < 36.4 mg/dL were found to have a 6-fold increased risk of decompensation (OR: 6.072, 95% CI 2.39-15.39). In contrast, total cholesterol, LDL-C, and TG did not show any correlation with known prognostic scoring systems and clinical outcomes in this study. However, HDL-C did not show any association with the development of ACLF on follow-up. In a recent study by Trieb and colleagues, both HDL-C and ApoA1 were found to be robust predictors of decompensation and short-term mortality. The area under ROC for acute decompensation was found to be 0.782 (*p* value < 0.001). They also found a significant association of HDL-C with the incidence of ACLF in that study. Optimal cut-off values obtained for HDL-C as a marker of short-term mortality was 17 mg/dL [40]. In another retrospective study by Habib et al., HDL-C, but not cholesterol or LDL-C, was strongly associated with bilirubin, albumin, MELD, and INR. By logistic regression analysis, HDL-C was identified as an independent prognostic factor for 6- and 12-month mortality in noncholestatic cirrhosis [42].

There are a few limitations to the present study. Firstly, total HDL-C levels were used in this study. HDL particles, each of which has distinct functions, could potentially delineate the mechanism of immune-modulatory actions of this molecule and inform future research in this area. Secondly, functionality assessment of HDL was not done in the study. Functionality assessment using cholesterol efflux capacity could provide a multidimensional perspective on the role of HDL-C in the pathogenesis of decompensation in patients with CLD. Lastly, the relationship between HDL-C and portal hypertension has not been assessed in this study. Portal hypertension has been shown to be intimately related to decompensation in patients with CLD [10, 43]. Prospective studies that can correlate HDL-C with HVPG levels could strengthen the association of HDL-C with the risk of decompensation and clinical outcome.

## 5. Conclusion

Serum HDL-C is a powerful predictor for decompensation in patients with CLD. It has shown an independent association with decompensation events in this study, and patients with HDL-C levels < 36.4 mg/dl were found to be six times more likely to have decompensation events within 1 year. HDL-C level was also found to have a linear inverse relationship with inflammatory markers. These findings add to a growing body of evidence that highlights the role of SI in the pathogenesis of decompensation of CLD and development of ACLF. Moreover, HDL-C is an easily available serum marker that can serve as a useful addition to the growing pantheon of inflammatory markers that help in prognostication. HDL-C can potentially become an integral component of management protocols and help to prioritise dCLD patients for liver transplantation. Nevertheless, larger prospective, multicentric studies combining key inflammatory markers are warranted before recommending the routine use of HDL-C in clinical practice.

## Figures and Tables

**Table 1:** Distribution of variables among patients with decompensation events on follow-up as compared to patients with decompensation.

Baseline characteristics of the population	Total *N* = 170
Mean age (mean ± SD, years)	60 ± 11.5
Gender distribution (*n* (%))	
Male	147 (86.5)
Female	23 (13.5)
Median duration of CLD at recruitment (days)	1121
Etiology of CLD (*n* (%))	
Alcohol	74 (43.5)
Viral hepatitis	11 (6.4)
Cryptogenic	85 (50)
Comorbidities (*n* (%))	
Diabetes mellitus	110 (64.7)
Systemic hypertension	51 (30)
Coronary artery disease	19 (11.2)
Chronic kidney disease	30 (17.6)
Child status (*n* (%))	
Child A	37 (21.8)
Child B	32 (18.8)
Child C	101 (59.4)
Mean MELD score (mean ± SD)	20 ± 7.04
Past history of complications of CLD (*n* (%))	
Varices on endoscopy	126 (74.11)
Ascites	95 (55.88)
Hepatic encephalopathy	44 (25.88)
Variceal bleed	41 (24.11)
Laboratory parameters: (mean ± SD)	
Total cholesterol	119.16 ± 49.92
High-density lipoprotein	34.44 ± 16.57
Low-density lipoprotein	73.7 ± 38.32
Total leucocyte count	6.59 ± 4.24
Platelet count	111.4 ± 69.07
Creatinine	1.15 ± 0.724
Sodium	134.85 ± 4.86
Potassium	4.16 ± 0.57
Albumin	3.21 ± 0.724
INR	1.71 ± 0.74
C-reactive protein	15.02 ± 23.4
Neutrophil-lymphocyte ratio (NLR)	3.63 ± 3.93
Total decompensation events at follow-up (*n* (%))	97 (57.05)
Ascites	67 (69.07)
Hepatic encephalopathy	41 (42.26)
Hepatorenal syndrome	45 (46.39)
Variceal bleed	20 (20.61)
Mortality (*n* (%))	43 (25.3)

Abbreviations: CLD: chronic liver disease; MELD score: model for end-stage liver disease; INR: international normalized ratio.

**Table 2:** Factors associated with risk of decompensation on follow-up in patients with cirrhosis of liver.

	Without decompensation events *n* = 73	With decompensation events *n* = 97	*p* value
Mean age (mean ± SD, years)	65 ± 11.23	57 ± 10.57	**<**0.001^∗^
Etiology of CLD (*n* (%))			
Alcohol	31 (42.5)	43 (44.3)	
Viral hepatitis	9 (12.3)	2 (2.06)	0.043^∗^
Cryptogenic	33 (45.2)	52 (53.6)	
Comorbidities (*n* (%))			
Diabetes mellitus	56 (76.7)	54 (55.7)	0.004^∗^
Systemic hypertension	26 (35.6)	25 (25.8)	0.166
Coronary artery disease	11 (15.1)	8 (8.2)	0.162
Chronic kidney disease	17 (23.3)	13 (13.4)	0.094
Child status (*n* (%))			
Child A	31 (42.5)	6 (6.2)	
Child B	15 (20.5)	17 (17.5)	**<**0.001^∗^
Child C	27 (37)	74 (76.3)	
Mean MELD score (mean ± SD)	17.07 ± 6.5	23.03 ± 6.29	**<**0.001^∗^
Past history of complications of CLD (*n* (%))			
Varices on endoscopy	57 (78.1)	69 (71.1)	0.306
Ascites	1 (1.4)	66 (68)	**<**0.001^∗^
Hepatic encephalopathy	0 (0)	41 (42.3)	**<**0.001^∗^
Hepatorenal syndrome	0 (0)	45 (46.4)	**<**0.001^∗^
Variceal bleed	23 (31.5)	18 (18.6)	0.006^∗^
Laboratory parameters (mean ± SD)			
Total cholesterol	144.38 ± 43.97	100.19 ± 45.71	0.258
High-density lipoprotein	43.56 ± 13.94	27.58 ± 15.07	**<**0.001^∗^
Low-density lipoprotein	89.89 ± 37.22	61.54 ± 34.61	0.608
Total leucocyte count	5.65 ± 1.85	7.30 ± 5.28	0.048^∗^
Platelet count	122.79 ± 65.94	102.85 ± 70.46	0.033^∗^
Creatinine	1.19 ± 0.75	1.12 ± 0.70	0.850
Sodium	136.61 ± 3.78	133.55 ± 5.16	0.029^∗^
Potassium	4.25 ± 0.54	4.08 ± 0.58	0.427
Albumin	3.52 ± 0.74	2.97 ± 0.61	**<**0.001^∗^
INR	1.34 ± 0.42	1.97 ± 0.81	**<**0.001^∗^
C-reactive protein	11.31 ± 19.60	17.66 ± 25.56	0.080
Neutrophil-lymphocyte ratio (NLR)	2.48 ± 1.53	4.50 ± 4.86	0.003^∗^

^∗^Statistically significant (<0.05). Abbreviations: CLD: chronic liver disease; MELD score: model for end-stage liver disease; INR: international normalized ratio.

## Data Availability

No additional data are available.
